# Diethyl [4-(2,2′:6′,2′′-terpyridine-4′-yl)phen­yl]phospho­nate

**DOI:** 10.1107/S1600536813031541

**Published:** 2013-11-27

**Authors:** Jan Chyba, Marek Necas, Jiri Pinkas

**Affiliations:** a Department of Chemistry, Faculty of Science, Masaryk University, Kotlarska 2, CZ-61137 Brno, Czech Republic; b Central European Institute of Technology (CEITEC), Masaryk University, 625 00 Brno, Czech Republic

## Abstract

The title compound, C_25_H_24_N_3_O_3_P, was obtained by catalytic phospho­nation of 4′-(4-bromphen­yl)-2,2′:6′,2′′-terpyridine. The terpyridine moiety is nearly planar, the dihedral angles between the central and the outer rings being 4.06 (9) and 5.39 (9)°. The N atoms in the two pyridine rings are oriented nearly anti­periplanar to that of the central ring. The benzene ring is rotated out of the plane of the central ring of the terpyridine unit by 34.65 (6)°.

## Related literature

Terpyridines (Heller & Schubert, 2003 ▶) are frequently employed as tridentate chelating ligands for transition and rare earth metals forming very stable square planar mono- (Eryazici *et al.*, 2008 ▶) or octa­hedral bis-complexes (Constable, 2007 ▶, 2008 ▶). For related symmetrical 4′-substituted terpyridine derivatives, see: Hofmeier & Schubert (2004 ▶); Andres & Schubert (2004 ▶).
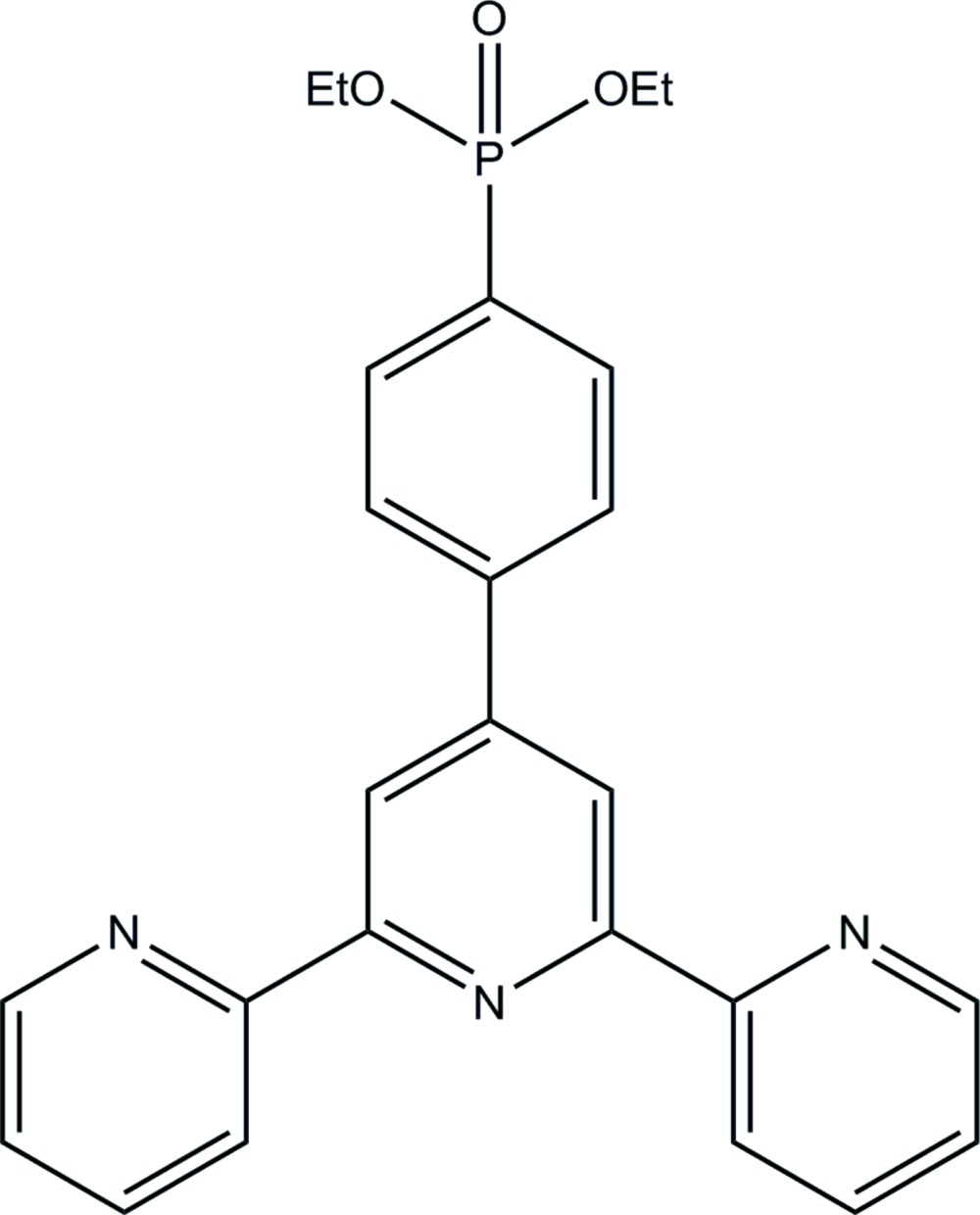


## Experimental

### 

#### Crystal data

C_25_H_24_N_3_O_3_P
*M* *_r_* = 445.44
Monoclinic, 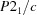

*a* = 12.5290 (4) Å
*b* = 13.0264 (4) Å
*c* = 14.5681 (5) Å
β = 111.674 (3)°
*V* = 2209.53 (12) Å^3^
*Z* = 4
Mo *K*α radiation
μ = 0.16 mm^−1^
*T* = 120 K
0.40 × 0.40 × 0.30 mm

#### Data collection

Oxford Diffraction Xcalibur (Sapphire2) diffractometer
Absorption correction: multi-scan (*CrysAlis RED*; Oxford Diffraction, 2009 ▶) *T*_min_ = 0.957, *T*_max_ = 1.000
23115 measured reflections
3886 independent reflections
2809 reflections with *I* > 2σ(*I*)
*R*_int_ = 0.027

#### Refinement

*R*[*F* ^2^ > 2σ(*F* ^2^)] = 0.030
*wR*(*F* ^2^) = 0.075
*S* = 1.02
3886 reflections
291 parameters
H-atom parameters constrained
Δρ_max_ = 0.26 e Å^−3^
Δρ_min_ = −0.36 e Å^−3^

### 

Data collection: *CrysAlis CCD* (Oxford Diffraction, 2009 ▶); cell refinement: *CrysAlis RED* (Oxford Diffraction, 2009 ▶); data reduction: *CrysAlis RED*; program(s) used to solve structure: *SHELXS97* (Sheldrick, 2008 ▶); program(s) used to refine structure: *SHELXL97* (Sheldrick, 2008 ▶); molecular graphics: *SHELXTL* (Sheldrick, 2008 ▶); software used to prepare material for publication: *SHELXL97*.

## Supplementary Material

Crystal structure: contains datablock(s) I. DOI: 10.1107/S1600536813031541/nc2320sup1.cif

Structure factors: contains datablock(s) I. DOI: 10.1107/S1600536813031541/nc2320Isup2.hkl

Supplementary material file. DOI: 10.1107/S1600536813031541/nc2320Isup3.cml

Additional supplementary materials:  crystallographic information; 3D view; checkCIF report

## Figures and Tables

**Fig. 1.**
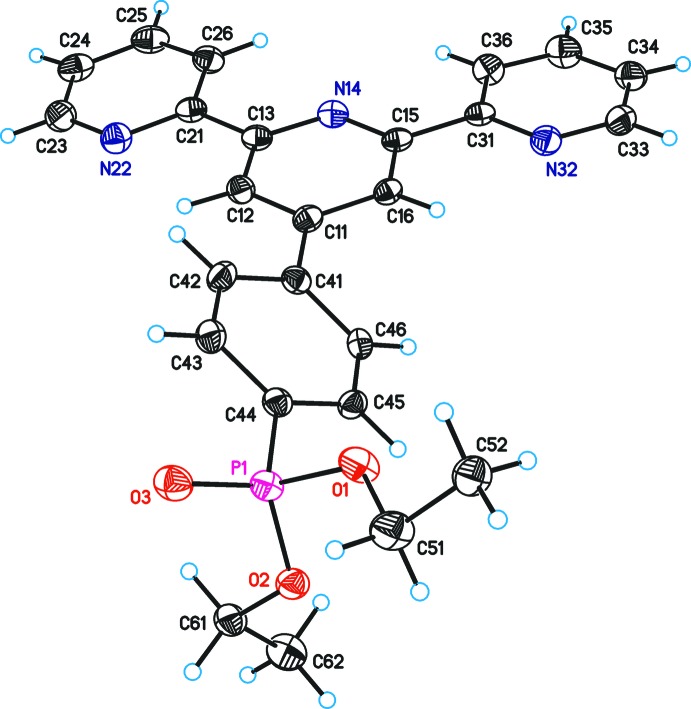
: ORTEP of the asymmetric unit with atoms represented as 50% probability ellipsoids.

## supplementary crystallographic information

### 1. Comment

The title compound was prepared as a precursor for our study of molecular aluminophosphate building blocks possessing metal-coordinating groups. The molecule presents the usual conformation of nitrogen atoms in the two pyridine rings as nearly antiperiplanar oriented to that of the central ring of the terpyridine moiety (Fig. 1). The P=O bond adopts an essentially *syn* conformation with the plane of the attached ring. The torsion angle O3–P1–C44–C43 is 6.93 (14)°. Thus, the ethyl groups are conveniently located below and above the plane of the ring. All four aromatic rings are planar, with r.m.s. deviations not exceeding 0.0066 Å.

### 2. Experimental

To a mixture of diethylphosphite (C_2_H_5_O)_2_P(O)H (1.1 cm^3^, 8.5 mmol, Fluka), triethylamine (1.3 cm^3^, 9.4 mmol) and tetrakis(triphenylphosphine)palladium (0.337 g, 0.292 mmol), a solution of 4'-(4-bromophenyl)-2,2':6',2''-terpyridine (2.268 g, 5.841 mmol) in toluene (10 cm^3^) was added at 50 °C. The reaction mixture was stirred for 3.5 h at 90 °C and a white solid triethylammonium bromide precipitated. After cooling, the solid was filtered off and all volatile components were removed from the filtrate under vacuum. The final product was isolated by liquid chromatography (stationary phase: silicagel, mobile phase: petrolether, ethylacetate, triethylamine – 1:1:0.04). Yield 46.7% (1.215 g). M>.p. 122 °C. Single crystals suitable for X-ray diffraction analysis were obtained by a slow evaporation of a CHCl_3_ solution.

NMR spectra were obtained on a Bruker AVANCE DRX 300 MHz spectrometer.

^1^H NMR (300.13 MHz, CDCl_3_): δ (p.p.m.) = 1.34 (t, 6H); 4.15 (m, 4H); 7.34 (m, 2H); 7.86 (m, 2H); 7.94 (AA'XX', m, 2H); 8.12 (AA'XX', m, 2H); 8.65 (m, 2H); 8.71 (m, 2H); 8.73 (s, 2H). ^13^C{^1^H} NMR (75.48 MHz, CD_2_Cl_2_): δ (p.p.m.) = 16.8 (d, ^3^J_PC_ = 6.0 Hz); 62.7 (d, ^2^J_PC_ = 5.5 Hz); 119.1; 121.6; 124.4; 127.7 (d, ^2^J_PC_ = 15.4 Hz); 129.9 (d, ^1^J_PC_ = 188.2 Hz); 132.8 (d, ^3^J_PC_ = 9.8 Hz); 137.2; 142.8 (d, ^4^J_PC_ = 3.2 Hz); 149.2; 149.6; 156.2; 156.6. ^31^P{^1^H} NMR (121.50 MHz, CD_2_Cl_2_): δ (p.p.m.) = 18.8 (s).

Mass spectra were recorded on a quadrupole mass spectrometer TRIO 1000 series II, Finnigan MAT, Fisons Instruments, with resolution of 1 *m/z* in the range 0–1000 *m/z*. Mass spectrum (EI) *m/z* 445 (*M*^+^).

IR spectra were measured on a IFS 28 Bruker spectrometer on samples prepared as KBr pellets (1.5 mg in 100 mg of KBr). IR (KBr pellet, cm^-1^): ν 3051 w, 2988 m, 2941 w, 2895 w, 1583 s, 1567 m, 1535 m, 1468 m, 1382 m, 1245 vs, 1163 m, 1132 m, 1058 s, 1024 vs, 972 vs, 958 s, 839 w, 794 s, 774 m, 747 m, 669 m, 622 w, 575 s, 533 m.

### 3. Refinement

All H atoms were placed at calculated positions and were refined as riding with their *U*_iso_ set to either 1.2*U*_eq_ or 1.5*U*_eq_ (methyl) of the respective carrier atoms. The methyl H atoms were allowed to rotate about the C—C bond.

### Crystal data

**Table:**

C_25_H_24_N_3_O_3_P	*F*(000) = 936
*M**_r_* = 445.44	*D*_x_ = 1.339 Mg m^−^^3^
Monoclinic, *P*2_1_/*c*	Mo *K*α radiation, λ = 0.71073 Å
Hall symbol: -P 2ybc	Cell parameters from 25622 reflections
*a* = 12.5290 (4) Å	θ = 2.9–27.1°
*b* = 13.0264 (4) Å	µ = 0.16 mm^−^^1^
*c* = 14.5681 (5) Å	*T* = 120 K
β = 111.674 (3)°	Prism, colourless
*V* = 2209.53 (12) Å^3^	0.40 × 0.40 × 0.30 mm
*Z* = 4	

### Data collection

**Table:**

Oxford Diffraction Xcalibur (Sapphire2) diffractometer	3886 independent reflections
Radiation source: Enhance (Mo) X-ray Source	2809 reflections with *I* > 2σ(*I*)
Graphite monochromator	*R*_int_ = 0.027
Detector resolution: 8.4353 pixels mm^-1^	θ_max_ = 25.0°, θ_min_ = 2.9°
ω scan	*h* = −14→14
Absorption correction: multi-scan (*CrysAlis RED*; Oxford Diffraction, 2009)	*k* = −15→15
*T*_min_ = 0.957, *T*_max_ = 1.000	*l* = −17→17
23115 measured reflections	

### Refinement

**Table:**

Refinement on *F*^2^	Primary atom site location: structure-invariant direct methods
Least-squares matrix: full	Secondary atom site location: difference Fourier map
*R*[*F*^2^ > 2σ(*F*^2^)] = 0.030	Hydrogen site location: inferred from neighbouring sites
*wR*(*F*^2^) = 0.075	H-atom parameters constrained
*S* = 1.02	*w* = 1/[σ^2^(*F*_o_^2^) + (0.0428*P*)^2^] where *P* = (*F*_o_^2^ + 2*F*_c_^2^)/3
3886 reflections	(Δ/σ)_max_ = 0.001
291 parameters	Δρ_max_ = 0.26 e Å^−^^3^
0 restraints	Δρ_min_ = −0.36 e Å^−^^3^

### Special details

**Table:**

Geometry. All e.s.d.'s (except the e.s.d. in the dihedral angle between two l.s. planes) are estimated using the full covariance matrix. The cell e.s.d.'s are taken into account individually in the estimation of e.s.d.'s in distances, angles and torsion angles; correlations between e.s.d.'s in cell parameters are only used when they are defined by crystal symmetry. An approximate (isotropic) treatment of cell e.s.d.'s is used for estimating e.s.d.'s involving l.s. planes.
Refinement. Refinement of *F*^2^ against ALL reflections. The weighted *R*-factor *wR* and goodness of fit *S* are based on *F*^2^, conventional *R*-factors *R* are based on *F*, with *F* set to zero for negative *F*^2^. The threshold expression of *F*^2^ > σ(*F*^2^) is used only for calculating *R*-factors(gt) *etc*. and is not relevant to the choice of reflections for refinement. *R*-factors based on *F*^2^ are statistically about twice as large as those based on *F*, and *R*- factors based on ALL data will be even larger.

### Fractional atomic coordinates and isotropic or equivalent isotropic displacement parameters (Å^2^)

**Table:**

	*x*	*y*	*z*	*U*_iso_*/*U*_eq_	
P1	0.50120 (4)	0.72076 (3)	−0.00200 (3)	0.02069 (12)	
O1	0.46530 (9)	0.83650 (7)	−0.02492 (7)	0.0275 (3)	
O2	0.39364 (8)	0.67611 (7)	0.01687 (7)	0.0228 (3)	
O3	0.53164 (9)	0.66516 (8)	−0.07662 (7)	0.0272 (3)	
C11	0.89136 (13)	0.75905 (11)	0.39920 (11)	0.0200 (3)	
C12	0.98093 (13)	0.68836 (11)	0.43599 (11)	0.0209 (4)	
H12	0.9839	0.6300	0.3979	0.025*	
C13	1.06611 (13)	0.70391 (11)	0.52914 (11)	0.0207 (4)	
N14	1.06504 (10)	0.78575 (9)	0.58592 (9)	0.0217 (3)	
C15	0.97864 (13)	0.85442 (11)	0.54983 (11)	0.0190 (3)	
C16	0.89195 (13)	0.84309 (11)	0.45779 (10)	0.0185 (3)	
H16	0.8328	0.8931	0.4348	0.022*	
C21	1.16156 (13)	0.62884 (11)	0.57298 (11)	0.0213 (4)	
N22	1.15825 (11)	0.54273 (10)	0.52078 (9)	0.0263 (3)	
C23	1.24131 (14)	0.47314 (12)	0.56195 (12)	0.0291 (4)	
H23	1.2398	0.4117	0.5263	0.035*	
C24	1.32901 (14)	0.48532 (12)	0.65303 (12)	0.0291 (4)	
H24	1.3855	0.4335	0.6791	0.035*	
C25	1.33247 (14)	0.57469 (13)	0.70507 (12)	0.0303 (4)	
H25	1.3919	0.5859	0.7675	0.036*	
C26	1.24803 (13)	0.64732 (12)	0.66465 (11)	0.0252 (4)	
H26	1.2487	0.7095	0.6990	0.030*	
C31	0.97814 (13)	0.94306 (11)	0.61461 (11)	0.0194 (4)	
N32	0.89127 (10)	1.01071 (10)	0.57719 (9)	0.0257 (3)	
C33	0.88630 (14)	1.08950 (12)	0.63477 (12)	0.0286 (4)	
H33	0.8261	1.1381	0.6087	0.034*	
C34	0.96454 (14)	1.10380 (12)	0.73049 (12)	0.0281 (4)	
H34	0.9577	1.1602	0.7693	0.034*	
C35	1.05227 (15)	1.03371 (12)	0.76736 (12)	0.0310 (4)	
H35	1.1073	1.0410	0.8326	0.037*	
C36	1.05986 (14)	0.95269 (11)	0.70898 (11)	0.0251 (4)	
H36	1.1204	0.9042	0.7333	0.030*	
C41	0.79611 (13)	0.74623 (10)	0.30129 (10)	0.0195 (3)	
C42	0.81495 (13)	0.70342 (11)	0.22064 (11)	0.0237 (4)	
H42	0.8895	0.6798	0.2285	0.028*	
C43	0.72622 (13)	0.69490 (11)	0.12923 (11)	0.0236 (4)	
H43	0.7408	0.6657	0.0753	0.028*	
C44	0.61595 (13)	0.72881 (10)	0.11592 (11)	0.0189 (3)	
C45	0.59641 (13)	0.76988 (11)	0.19754 (11)	0.0199 (3)	
H45	0.5215	0.7921	0.1902	0.024*	
C46	0.68515 (13)	0.77839 (10)	0.28863 (11)	0.0192 (3)	
H46	0.6704	0.8064	0.3430	0.023*	
C51	0.36229 (14)	0.86636 (12)	−0.10752 (12)	0.0311 (4)	
H51A	0.2930	0.8506	−0.0927	0.037*	
H51B	0.3574	0.8284	−0.1678	0.037*	
C52	0.36991 (14)	0.97985 (11)	−0.12287 (12)	0.0276 (4)	
H52A	0.3027	1.0020	−0.1796	0.041*	
H52B	0.4399	0.9948	−0.1355	0.041*	
H52C	0.3720	1.0167	−0.0636	0.041*	
C61	0.39903 (14)	0.56972 (11)	0.05077 (12)	0.0241 (4)	
H61A	0.4794	0.5449	0.0748	0.029*	
H61B	0.3522	0.5251	−0.0044	0.029*	
C62	0.35357 (15)	0.56613 (12)	0.13283 (11)	0.0318 (4)	
H62A	0.3562	0.4953	0.1563	0.048*	
H62B	0.2740	0.5907	0.1083	0.048*	
H62C	0.4008	0.6100	0.1874	0.048*	

### Atomic displacement parameters (Å^2^)

**Table:**

	*U*^11^	*U*^22^	*U*^33^	*U*^12^	*U*^13^	*U*^23^
P1	0.0205 (2)	0.0200 (2)	0.0200 (2)	−0.00170 (18)	0.00562 (18)	0.00025 (18)
O1	0.0267 (7)	0.0211 (6)	0.0264 (6)	−0.0013 (5)	0.0002 (5)	0.0043 (5)
O2	0.0203 (6)	0.0203 (5)	0.0264 (6)	−0.0003 (5)	0.0069 (5)	0.0015 (5)
O3	0.0278 (7)	0.0316 (6)	0.0216 (6)	−0.0048 (5)	0.0086 (5)	−0.0032 (5)
C11	0.0192 (9)	0.0223 (8)	0.0202 (9)	−0.0005 (7)	0.0093 (7)	0.0025 (7)
C12	0.0212 (9)	0.0224 (8)	0.0189 (9)	−0.0014 (7)	0.0072 (8)	−0.0026 (7)
C13	0.0201 (9)	0.0222 (8)	0.0202 (9)	−0.0031 (7)	0.0079 (8)	0.0019 (7)
N14	0.0218 (8)	0.0241 (7)	0.0189 (7)	−0.0020 (6)	0.0072 (6)	0.0017 (6)
C15	0.0164 (9)	0.0224 (8)	0.0184 (8)	−0.0025 (7)	0.0069 (7)	0.0034 (7)
C16	0.0163 (9)	0.0208 (8)	0.0182 (8)	0.0013 (6)	0.0062 (7)	0.0032 (6)
C21	0.0192 (9)	0.0248 (8)	0.0197 (9)	−0.0030 (7)	0.0071 (8)	0.0022 (7)
N22	0.0253 (8)	0.0261 (7)	0.0269 (8)	0.0017 (6)	0.0087 (7)	0.0025 (6)
C23	0.0293 (11)	0.0248 (9)	0.0349 (10)	0.0012 (8)	0.0139 (9)	0.0029 (7)
C24	0.0203 (10)	0.0293 (9)	0.0350 (10)	0.0028 (7)	0.0070 (8)	0.0124 (8)
C25	0.0211 (10)	0.0368 (10)	0.0271 (10)	−0.0033 (8)	0.0021 (8)	0.0060 (8)
C26	0.0226 (10)	0.0262 (9)	0.0236 (9)	−0.0025 (7)	0.0048 (8)	0.0001 (7)
C31	0.0166 (9)	0.0228 (9)	0.0194 (9)	−0.0028 (7)	0.0073 (8)	0.0028 (7)
N32	0.0215 (8)	0.0285 (7)	0.0246 (8)	−0.0009 (6)	0.0057 (6)	−0.0042 (6)
C33	0.0219 (10)	0.0310 (9)	0.0323 (10)	−0.0005 (8)	0.0093 (8)	−0.0069 (8)
C34	0.0291 (10)	0.0322 (9)	0.0263 (10)	−0.0074 (8)	0.0141 (9)	−0.0106 (7)
C35	0.0308 (11)	0.0379 (10)	0.0195 (9)	−0.0040 (8)	0.0038 (8)	−0.0041 (8)
C36	0.0243 (10)	0.0276 (9)	0.0191 (9)	0.0010 (7)	0.0028 (8)	0.0013 (7)
C41	0.0199 (9)	0.0171 (8)	0.0198 (9)	−0.0010 (6)	0.0054 (8)	0.0011 (6)
C42	0.0167 (9)	0.0273 (9)	0.0261 (10)	0.0034 (7)	0.0066 (8)	−0.0026 (7)
C43	0.0246 (10)	0.0257 (9)	0.0215 (9)	−0.0001 (7)	0.0098 (8)	−0.0044 (7)
C44	0.0197 (9)	0.0152 (7)	0.0216 (9)	−0.0007 (7)	0.0072 (7)	0.0020 (6)
C45	0.0165 (9)	0.0192 (8)	0.0231 (9)	0.0022 (7)	0.0063 (8)	0.0022 (7)
C46	0.0227 (9)	0.0156 (7)	0.0205 (9)	0.0016 (7)	0.0093 (8)	−0.0009 (6)
C51	0.0262 (10)	0.0300 (9)	0.0292 (10)	0.0014 (8)	0.0011 (8)	0.0062 (8)
C52	0.0260 (10)	0.0269 (9)	0.0275 (9)	0.0029 (7)	0.0070 (8)	0.0034 (7)
C61	0.0239 (10)	0.0180 (8)	0.0294 (9)	−0.0006 (7)	0.0087 (8)	0.0017 (7)
C62	0.0351 (11)	0.0312 (9)	0.0287 (10)	−0.0044 (8)	0.0115 (9)	0.0024 (8)

### Geometric parameters (Å, º)

**Table:**

P1—O3	1.4691 (10)	C33—C34	1.389 (2)
P1—O1	1.5734 (10)	C33—H33	0.9500
P1—O2	1.5822 (10)	C34—C35	1.376 (2)
P1—C44	1.7902 (16)	C34—H34	0.9500
O1—C51	1.4553 (17)	C35—C36	1.381 (2)
O2—C61	1.4644 (16)	C35—H35	0.9500
C11—C16	1.3865 (19)	C36—H36	0.9500
C11—C12	1.397 (2)	C41—C42	1.3972 (19)
C11—C41	1.493 (2)	C41—C46	1.397 (2)
C12—C13	1.397 (2)	C42—C43	1.389 (2)
C12—H12	0.9500	C42—H42	0.9500
C13—N14	1.3524 (18)	C43—C44	1.394 (2)
C13—C21	1.493 (2)	C43—H43	0.9500
N14—C15	1.3520 (18)	C44—C45	1.4053 (19)
C15—C16	1.387 (2)	C45—C46	1.386 (2)
C15—C31	1.4928 (19)	C45—H45	0.9500
C16—H16	0.9500	C46—H46	0.9500
C21—N22	1.3472 (18)	C51—C52	1.503 (2)
C21—C26	1.395 (2)	C51—H51A	0.9900
N22—C23	1.3429 (19)	C51—H51B	0.9900
C23—C24	1.384 (2)	C52—H52A	0.9800
C23—H23	0.9500	C52—H52B	0.9800
C24—C25	1.381 (2)	C52—H52C	0.9800
C24—H24	0.9500	C61—C62	1.503 (2)
C25—C26	1.379 (2)	C61—H61A	0.9900
C25—H25	0.9500	C61—H61B	0.9900
C26—H26	0.9500	C62—H62A	0.9800
C31—N32	1.3491 (18)	C62—H62B	0.9800
C31—C36	1.384 (2)	C62—H62C	0.9800
N32—C33	1.3414 (19)		
			
O3—P1—O1	116.57 (6)	C33—C34—H34	121.0
O3—P1—O2	114.70 (6)	C34—C35—C36	119.57 (15)
O1—P1—O2	101.19 (6)	C34—C35—H35	120.2
O3—P1—C44	113.77 (7)	C36—C35—H35	120.2
O1—P1—C44	102.29 (6)	C35—C36—C31	119.17 (15)
O2—P1—C44	106.77 (6)	C35—C36—H36	120.4
C51—O1—P1	121.89 (9)	C31—C36—H36	120.4
C61—O2—P1	118.05 (9)	C42—C41—C46	118.52 (14)
C16—C11—C12	117.61 (14)	C42—C41—C11	121.62 (14)
C16—C11—C41	119.81 (13)	C46—C41—C11	119.86 (13)
C12—C11—C41	122.57 (13)	C43—C42—C41	120.96 (14)
C11—C12—C13	119.64 (14)	C43—C42—H42	119.5
C11—C12—H12	120.2	C41—C42—H42	119.5
C13—C12—H12	120.2	C42—C43—C44	120.62 (14)
N14—C13—C12	122.12 (14)	C42—C43—H43	119.7
N14—C13—C21	116.23 (13)	C44—C43—H43	119.7
C12—C13—C21	121.62 (13)	C43—C44—C45	118.44 (14)
C15—N14—C13	118.11 (13)	C43—C44—P1	121.22 (11)
N14—C15—C16	122.29 (13)	C45—C44—P1	120.34 (12)
N14—C15—C31	117.10 (13)	C46—C45—C44	120.79 (14)
C16—C15—C31	120.59 (13)	C46—C45—H45	119.6
C11—C16—C15	120.22 (13)	C44—C45—H45	119.6
C11—C16—H16	119.9	C45—C46—C41	120.65 (13)
C15—C16—H16	119.9	C45—C46—H46	119.7
N22—C21—C26	122.35 (14)	C41—C46—H46	119.7
N22—C21—C13	116.81 (13)	O1—C51—C52	107.47 (12)
C26—C21—C13	120.83 (14)	O1—C51—H51A	110.2
C23—N22—C21	116.92 (13)	C52—C51—H51A	110.2
N22—C23—C24	124.10 (15)	O1—C51—H51B	110.2
N22—C23—H23	117.9	C52—C51—H51B	110.2
C24—C23—H23	117.9	H51A—C51—H51B	108.5
C25—C24—C23	118.39 (15)	C51—C52—H52A	109.5
C25—C24—H24	120.8	C51—C52—H52B	109.5
C23—C24—H24	120.8	H52A—C52—H52B	109.5
C26—C25—C24	118.71 (15)	C51—C52—H52C	109.5
C26—C25—H25	120.6	H52A—C52—H52C	109.5
C24—C25—H25	120.6	H52B—C52—H52C	109.5
C25—C26—C21	119.51 (15)	O2—C61—C62	108.30 (12)
C25—C26—H26	120.2	O2—C61—H61A	110.0
C21—C26—H26	120.2	C62—C61—H61A	110.0
N32—C31—C36	122.19 (14)	O2—C61—H61B	110.0
N32—C31—C15	116.16 (13)	C62—C61—H61B	110.0
C36—C31—C15	121.60 (13)	H61A—C61—H61B	108.4
C33—N32—C31	117.64 (13)	C61—C62—H62A	109.5
N32—C33—C34	123.48 (15)	C61—C62—H62B	109.5
N32—C33—H33	118.3	H62A—C62—H62B	109.5
C34—C33—H33	118.3	C61—C62—H62C	109.5
C35—C34—C33	117.94 (15)	H62A—C62—H62C	109.5
C35—C34—H34	121.0	H62B—C62—H62C	109.5
			
O3—P1—O1—C51	64.82 (12)	N14—C15—C31—C36	−2.4 (2)
O2—P1—O1—C51	−60.30 (12)	C16—C15—C31—C36	176.11 (13)
C44—P1—O1—C51	−170.42 (11)	C36—C31—N32—C33	0.3 (2)
O3—P1—O2—C61	59.59 (11)	C15—C31—N32—C33	177.95 (12)
O1—P1—O2—C61	−174.05 (10)	C31—N32—C33—C34	−0.9 (2)
C44—P1—O2—C61	−67.41 (11)	N32—C33—C34—C35	0.7 (2)
C16—C11—C12—C13	0.5 (2)	C33—C34—C35—C36	0.1 (2)
C41—C11—C12—C13	−178.66 (13)	C34—C35—C36—C31	−0.6 (2)
C11—C12—C13—N14	0.1 (2)	N32—C31—C36—C35	0.4 (2)
C11—C12—C13—C21	178.06 (13)	C15—C31—C36—C35	−177.07 (14)
C12—C13—N14—C15	−0.6 (2)	C16—C11—C41—C42	145.39 (14)
C21—C13—N14—C15	−178.60 (12)	C12—C11—C41—C42	−35.5 (2)
C13—N14—C15—C16	0.4 (2)	C16—C11—C41—C46	−33.99 (19)
C13—N14—C15—C31	178.91 (12)	C12—C11—C41—C46	145.12 (14)
C12—C11—C16—C15	−0.6 (2)	C46—C41—C42—C43	1.4 (2)
C41—C11—C16—C15	178.51 (13)	C11—C41—C42—C43	−177.99 (13)
N14—C15—C16—C11	0.2 (2)	C41—C42—C43—C44	−0.1 (2)
C31—C15—C16—C11	−178.24 (12)	C42—C43—C44—C45	−1.2 (2)
N14—C13—C21—N22	174.58 (12)	C42—C43—C44—P1	178.75 (11)
C12—C13—C21—N22	−3.5 (2)	O3—P1—C44—C43	6.93 (14)
N14—C13—C21—C26	−4.2 (2)	O1—P1—C44—C43	−119.67 (12)
C12—C13—C21—C26	177.82 (13)	O2—P1—C44—C43	134.48 (11)
C26—C21—N22—C23	1.4 (2)	O3—P1—C44—C45	−173.13 (11)
C13—C21—N22—C23	−177.27 (12)	O1—P1—C44—C45	60.27 (12)
C21—N22—C23—C24	−0.6 (2)	O2—P1—C44—C45	−45.58 (13)
N22—C23—C24—C25	−0.5 (2)	C43—C44—C45—C46	1.3 (2)
C23—C24—C25—C26	0.6 (2)	P1—C44—C45—C46	−178.67 (11)
C24—C25—C26—C21	0.2 (2)	C44—C45—C46—C41	0.0 (2)
N22—C21—C26—C25	−1.3 (2)	C42—C41—C46—C45	−1.3 (2)
C13—C21—C26—C25	177.38 (14)	C11—C41—C46—C45	178.09 (13)
N14—C15—C31—N32	179.95 (12)	P1—O1—C51—C52	−166.13 (10)
C16—C15—C31—N32	−1.51 (19)	P1—O2—C61—C62	135.67 (11)
